# Chemical Profiling, Enzyme Inhibitory Activity and Antioxidant Capacity of South African Herbal Teas: *Buddleja saligna*, *Lippia javanica*, *L. scaberrima* and *Phyla dulcis*

**DOI:** 10.3390/antiox13101219

**Published:** 2024-10-10

**Authors:** Nélida Nina, Alberto Burgos-Edwards, Cristina Theoduloz, Satyajit Tripathy, Motlalepula Gilbert Matsabisa, Guillermo Schmeda-Hirschmann

**Affiliations:** 1 Laboratorio de Química de Productos Naturales, Instituto de Química de Recursos Naturales, Campus Lircay, Universidad de Talca, Talca 3480094, Chile; nnina@utalca.cl (N.N.); aburgos@utalca.cl (A.B.-E.); 2 Departamento de Fitoquímica, Facultad de Ciencias Químicas, Universidad Nacional de Asunción, Campus San Lorenzo P.O. Box 2169, Paraguay; 3 Laboratorio de Cultivo Celular, Facultad de Ciencias de la Salud, Campus Lircay, Universidad de Talca, Talca 3480094, Chile; ctheoduloz@gmail.com; 4 Department of Physiology and Allied Sciences, Amity Institute of Health Allied Sciences, Amity University, Noida 201301, Uttar Pradesh, India; stripathy@amity.edu; 5 Department of Pharmacology, Faculty of Health Sciences, School of Clinical Medicines, University of Free State, Bloemfontein 9300, South Africa

**Keywords:** South African herbal teas, glucosidase inhibition, phenylpropanoid glycosides, HPLC-MS/MS studies, Scrophulariaceae, Verbenaceae

## Abstract

Herbal teas are used in South Africa as digestives to lower glycaemia and for other indications. However, the chemical composition of the infusions and their effect on enzymes related to metabolic syndrome is poorly known. The composition of infusions and methanol extracts of *B. saligna* (Scrophulariaceae), *Lippia javanica*, *L. scaberrima*, and *Phyla dulcis* (Verbenaceae) was assessed and the effect of the infusions and extract was determined towards α-glucosidase, α-amylase, and pancreatic lipase. The commercial herbal products were extracted separately with MeOH or hot water to obtain the extract/infusion for comparative studies. Total phenolic, total flavonoid and antioxidant capacity were assessed. The fingerprints of the MeOH extracts and infusions were compared by HPLD-DAD. The extract constituents were tentatively identified by HPLC-MS/MS and NMR analyses. From the extracts/infusions, 57 compounds were identified, including iridoids, phenylpropanoid glycosides, flavonoids, and caffeic acid derivatives, among others. The MeOH extracts and infusions showed strong inhibition towards α-glucosidase with IC_50_ in the range of 0.13–0.84 µg/mL for the phenolic-enriched infusion extract (PEI) and 0.47–0.50 µg/mL for the MeOH extracts, respectively. The *P. dulcis* PEI showed higher inhibition towards α-glucosidase, and the MeOH extract of *L. scaberrima* was better than the PEI. At 100 µg/mL, the PEI from the four herbal teas reduces the activity of α-amylase by 23.03–28.61%, with *L. javanica* as the most active tea. Three of the species are high in phenylpropanoid glycosides, while *P. dulcis* contains rosmarinic acid. Some 26 compounds were identified in the infusion from *B. saligna*, 28 from *L. scaberrima*, and 21 from *P. dulcis*. Four of them are common in all the teas, namely decaffeoylverbascoside, verbascoside, isoverbascoside, and tuberonic acid hexoside. Ten compounds occur in two of the teas and seventeen, fifteen, and eleven compounds were detected only in *B. saligna*, *L. scaberrima*, and *P. dulcis*, respectively. Most of the compounds are reported for the first time from the crude drug infusions. The results give some support for the traditional use of herbal teas as digestives and/or indications for diabetes. The chemical fingerprints set the basis for quality control of the crude drugs, based on the main constituents and differential compounds occurring in the samples.

## 1. Introduction

Herbal teas are used worldwide as digestives and are a way to ingest bioactive compounds following local lore or commercial trends. The best-known South African herbal tea is rooibos (*Aspalathus linearis*) and the traditional use of the product is related to a glucose lowering effect, including inhibition of the enzyme α-glucosidase [1]. The dihydrochalcone *C*-glucoside aspalathin has been identified as the main bioactive compound from rooibos tea. Other South African herbal teas can be potential sources of enzyme inhibitors associated with metabolic syndrome.

Metabolic syndrome is the association of obesity, hyperlipidemia, hypertension, and insulin resistance and it precedes an increase in the risk of cardiovascular diseases and the onset of type-2 diabetes. Inhibition of enzymes associated with type-2 diabetes, including α-amylase and α-glucosidase, is an alternative to reducing postprandial hyperglycemia.

The enzymes pancreatic lipase, α-amylase, and α-glucosidase are responsible for the breakdown of lipids and carbohydrates, respectively. The inhibition of these enzymes delays the absorption of fatty acids and monosaccharides, which is an important strategy for treating postprandial hyperglycemia and obesity [2].

Herbal teas are sources of polyphenols that confer organoleptic properties such as color, astringency, bitterness, or flavor, and functional characteristics such as antioxidant activity, and prevention or reduction of metabolic disorders [1,3,4,5], among others [6].

In South Africa, medicinal and aromatic plants are cultivated to develop healthy herbal products with the aim of improving the wellbeing of local communities and the country’s economy. A recent work on commercialized South African herbal teas showed the effect of selected crude drug (herbal tea) infusions on different antioxidant assays, as well as on the inhibition of the enzyme cyclooxygenase and the proliferation of a human cell line [7]. According to the information on the single plants reported, traditional uses are linked mainly to the taste and digestive properties. *Buddleja saligna* is a medicinal plant used in infusion to control glycaemia and is therefore recommended for treating diabetes [7]. However, evidence is needed to assess the effect of the extract and infusion of the herbal teas on enzymes associated with metabolic syndrome, such as α-glucosidase, α-amylase, and pancreatic lipase. *Lippia javanica*, *L. scaberrima*, and *P. dulcis* are used in infusions as digestive, among other uses [7]. The plant parts used for the herbal teas are the leaves.

In the present work, four plant species used as sources of herbal teas were investigated. The samples comprise the Scrophulariaceae *Buddleja saligna* Willd., and the Verbenaceae *Lippia scaberrima* Sond., *Lippia javanica* (Burm.f.) Spreng., and *Phyla dulcis* (Trevir.) Moldenke. According to The Plant List (http://www.theplantlist.org, accessed on 2 October 2024), *Phyla dulcis* (Trevir.) Moldenke is a synonym of *Phyla scaberrima* (Juss. ex Pers.) Moldenke. In the Flora of Yucatan, Mexico, *Phyla dulcis* (Trevir.) Moldenke is a basonym of *Lippia dulcis* Trevir. (https://www.cicy.mx/sitios/flora%20digital/ficha_virtual.php?especie=2258, accessed on 2 October 2024).

Little is known of the polar metabolites occurring in the extracts or infusions of the selected plants. From the aerial parts of *L. javanica* and *L. scaberrima*, verbascoside and isoverbascoside were reported [8]. The antimycobacterial and adjuvant effects of *L. scaberrima* were described [9]. However, the polar constituents occurring in the infusions were not identified due to the detection method selected (G-MS).

The herb *Phyla dulcis* was rediscovered as the source of the sweet compound hernandulcin studied in ancient Aztec manuscripts [10]. The herb *Phyla dulcis* (synonyms: *Lippia dulcis* Trevir. and *Lippia dulcis* var. *mexicana* Wehmer) is native to Mexico and Central America. The plants selected for the present study are cultivated and commercialized as herbal teas in the southern part of Africa. Due to the scarce information available, identification of the constituents in the infusions and the potential effect on enzymes related to digestion and metabolic syndrome can be useful for consumers. Our hypothesis is that the infusions from the selected teas will show some effect on enzymes associated with metabolic syndrome and linked to sugar metabolism, such as α-glucosidase or α-amylase.

## 2. Materials and Methods

### 2.1. Herbal Teas

The herbal teas were commercial samples from Northwest and Eastern Cape provinces. Traditional uses and botanical identification were described by Matsabisa et al. [7]. Crude drugs included the Scrophulariaceae *Buddleja saligna* Willd., known under the common name ‘Gancair’, as well as the Verbenaceae *Lippia javanica* (Burm.f.) Spreng. (‘Zinibar’), *Lippia scaberrima* Sond. (‘Mosukujane’), and *Phyla dulcis* (Trevir.) Moldenke (‘Haw Haw’). Plant voucher specimens were deposited at Bolus Herbarium (BLFU) at University of the Free State, Bloemfontein, South Africa under the reference numbers MGM0015, MGM005, MGM012, and MGM0016 for *B. saligna*, *L. javanica*, *L. scaberrima*, and *P. dulcis*, respectively.

### 2.2. Extraction of Bioactive Compounds

The teas were produced using plant leaves. The teas were prepared by placing tea bags in boiling water. The powdered plant material contained in the bags ranged from 1 to 2.5 g/bag. The plant material was removed from the bags, weighed, and infused in 400 mL of boiled water. After 10 min at room temperature, the infusion was filtered and activated Amberlite XAD-7 (Supelco, France, commercialized by sigmaaldrich.com, 89555 Steinheim, Germany) was added and stirred for 20 min. Then, the Amberlite was filtered and washed with distilled water, and the retained compounds were desorbed with MeOH. The solution was taken to dryness under reduced pressure and then lyophilized to afford the phenolic-enriched infusions (PEI). On the other hand, methanol extracts were prepared from the leaves by placing the samples in distilled MeOH in a 1:40 plant material–solvent ratio under sonication for 10 min. The process was repeated three times. The combined extracts were filtered, taken to dryness, and lyophilized, yielding the MeOH extracts. The infusion represents the way the herbal teas are consumed. Extraction with MeOH allows us to obtain medium and polar compounds except the most polar constituents, such as sugars and inorganic salts. The MeOH extract is analyzed after being taken to dryness under reduced pressure and lyophilized to a solid powder. The water infusion extracts fewer medium and low polarity compounds, and more sugars and inorganic salts than MeOH. To enrich the infusions in phenolics and remove sugars and salts, infusions were treated with Amberlite to adsorb polar organic compounds for further analysis.

### 2.3. Total Phenolic, Total Flavonoid and Total Procyanidin Content

The Folin–Ciocalteu reagent was used to determine total phenolic (TP) content. The total flavonoid (TF) content was measured using the aluminum trichloride method [11]. The 4-dimethylaminocinnamaldehyde (DMAC) method was used to determine the total content of proanthocyanidin (PAC) in the MeOH extracts and infusions

### 2.4. Antioxidant Capacity Assays

For the antioxidant capacity study, complementary assays were used, as described in [11,12]. The positive control was the flavonol quercetin. For FRAP and ORAC, the results are expressed as µmol TE/g extract. The results for TEAC are given as µM TE/g extract and DPPH as SC_50_ (μg/mL). All determinations were carried out in triplicate.

### 2.5. Enzyme Inhibition Studies

The MeOH extracts and the PEI of infusions were assessed for inhibition of enzymes α-amylase, α-glucosidase, and pancreatic lipase, as described in previous work [11]. The inhibition of α-amylase was determined at 550 nm wavelength (WL) at the final sample concentration of 100 µg/mL, as described in [11]. For α-glucosidase, the inhibition was measured at 415 nm WL using a concentration of a sample ranging from 0.1 to 100 µg/mL [11]. The inhibition of porcine pancreatic lipase was determined as reported in [11], at 400 nm WL.

### 2.6. NMR Profiles

To get an insight into the chemical composition of the teas, some 15 mg of the lyophilized infusion were dissolved in DMSO-d6, and ^1^H-NMR spectra were recorded at 400 MHz. Two-dimensional NMR, ^13^C-NMR, and COSY experiments were performed to identify the main compounds. The spectral data were processed using the Mestre Nova x64 program (MestreLab Research Laboratories, Santiago de Compostela, Spain).

### 2.7. HPLC-DAD Analyses

Shimadzu HPLC equipment from Shimadzu Corporation (Kyoto, Japan) was used for the analyses. The equipment included an LC-20AT pump, a CTO-20 AC column oven, and a UV diode array detector (SPD-M20A). The software used was LabSolution software (Version 5.51). The column used was 250 mm × 4.6 mm, with 5 µm of particle size Inertsil ODS-4 RP-18 (GL Sciences Inc., Tokyo, Japan). The column was kept at 30 °C. A linear gradient of solvents was used for the HPLC analyses. The solvents were: (A) 0.1% formic acid in water and (B) 0.1% formic acid in acetonitrile (ACN). The gradient was: 0–15 min, 90–85% A; 15–20 min, 85% A; 20–25 min, 85–82% A; 25–75 min, 82–70% A; 75–78 min, 70% A; and 78–82 min, 70–90% A. The compounds eluted were monitored at 360 and 330 nm and UV spectra were measured in the range of 200–600 nm for chromophore characterization. Some 20 μL of a 5 mg/mL solution of the samples was injected and the flow rate was maintained at 0.4 mL/min.

### 2.8. HPLC-MS/MS Analyses

The mass spectra of the compounds were acquired through a Bruker Daltonik GmbH (Bremen, Germany) UHPLC/HPLC-DAD Bruker Elute LC system. The system was coupled in tandem with a Compact Q-TOF spectrometer. The software used for data analysis was the Compass DataAnalysis 4.4 software from Bruker Daltonik GmbH (Bremen, Germany). The column and precolumn used for chromatographic separations were as follows: Kromasil 100 5 C18, 5 µm particle size, 250 mm × 4.6 mm (Kromasil, Akzo Nobel, Bohus, Sweden), Nova-Pak, Waters Corp., Milford, CT, USA, C-18 Precolumn (22 × 3.9 mm, 4 μm particle size). Samples were dissolved in a mixture of ACN: formic acid 0.1% (1:1, *v*/*v*), and 20 µL of this solution were injected at 5 mg/mL for analysis. The same conditions previously described for HPLC-DAD analyses were employed for the chromatographic separation. The ionization was performed by electrospray ionization (ESI), using nitrogen as a nebulizer (9 L/min) and drying gas (4 Bar, 200 °C). The MS conditions were as follows: electrospray needle, −3500 V; compensating endplate, −500 V; separator cone 1, 56.0 V; separator cone 2, 6.0 V; output compensation capillary, 84.6 V; and output capillary, 140.6 V. The collision energy was 10–25 eV in stepping mode, auto MS/MS mode (4 precursor/cycle), 50–1500 *m*/*z* (scan 0.2 s centroid mode), using helium as a collision gas. Sodium formate (10% formic acid, 1 M) was employed for internal calibration. The compounds were tentatively identified by comparison of their mass spectra, fragmentation pattern, and UV profile.

### 2.9. Statistical Analyses

Statistical analyses were performed using GraphPad Prism version 7.00 for Windows (GraphPad Software, La Jolla, CA, USA). Differences between samples were tested using analysis of variance (ANOVA) one way, followed by Tukey’s multiple comparisons tests, and a *p* < 0.05 was considered statistically significant. All determinations were conducted in triplicate or quadruplicate, and the results were presented as mean ± standard deviation (SD).

## 3. Results

### 3.1. Extraction Yields

The extraction yields of the plant material contained in the tea bags were variable, according to the solvent used (MeOH or hot water) and the plant species (Table 1). The highest content of MeOH soluble was for *B. saligna* (23.11%), followed by *P. dulcis* (12.83%) and the two *Lippia* species (6.82–6.16%). The yield of the PEI of infusions was in the range of 3.95–5.01%, with higher extraction for *B. saligna* and *L. javanica* (5.01% and 4.91%, respectively). Infusions dissolve mainly water-soluble polar compounds while MeOH also dissolves less polar constituents, including chlorophyll and lipophilic substances. Therefore, the extraction yields of the crude drugs using MeOH were higher. However, the content of the different phenolics is lower as the extraction is less selective. The rationale in studying the composition of infusions is the fact that they better represent what is contained in the beverages taken by users. For better detection, the infusions were enriched in phenolics and other polar compounds using Amberlite XAD-7 resin, as described in Section 2.2.

### 3.2. Phenolics and Antioxidant Capacity

Enrichment of components from the infusion by Amberlite XAD to yield the PEI, increased the TP, TF, and TPA (*L. scaberrima*, *P. dulcis*) content, with higher activity in the DPPH discoloration assay, FRAP, ORAC, and TEAC values (except for the ORAC value of *B. saligna*) (Table 2). The best antioxidant capacity was observed for *P. dulcis* in FRAP and ORAC, while *B. saligna* was better in the TEAC measurements. When looking at the DPPH results, all extracts presented SC_50_ values in the range of 5.16–7.00 µg/mL. The TP content of the extracts was higher in the PEIs. The identity of the components plays a relevant role in the antioxidant capacity, measured by the ORAC assay, as can be observed by comparing the *B. saligna* PEI (29.29 g GAE/100 g PEI) with the best effect towards DPPH and TEAC, but with lower ORAC activity (Table 2).

### 3.3. Enzyme Inhibition

All extracts were active as α-glucosidase inhibitors, with IC_50_ values ranging from 0.13 to 0.84 µg/mL for the PEI and 0.47 to 0.50 µg/mL for the MeOH extracts, respectively. The *P. dulcis* PEI showed higher inhibition towards α-glucosidase, and the MeOH extract of *L. scaberrima* was better than the PEI (Table 3). At 100 µg/mL, the PEI from the four herbal teas reduced the activity of α-amylase by 23.14, 28.61, and 23.03% for *B. saligna*, *L. scaberrima*, and *P. dulcis*, respectively, while the most active tea was *L. javanica*, with an IC_50_ of 34.27 µg/mL. All MeOH extracts were inactive against pancreatic lipase at 50 µg/mL, but the PEI from *B. saligna* and *L. javanica* showed a mild effect, reducing the enzyme activity by 7.25 and 6.87%, respectively. None of the extracts showed an inhibition comparable to that of the reference compound Orlistat^®^ (Table 3).

### 3.4. NMR Analysis

**Figure 1:**
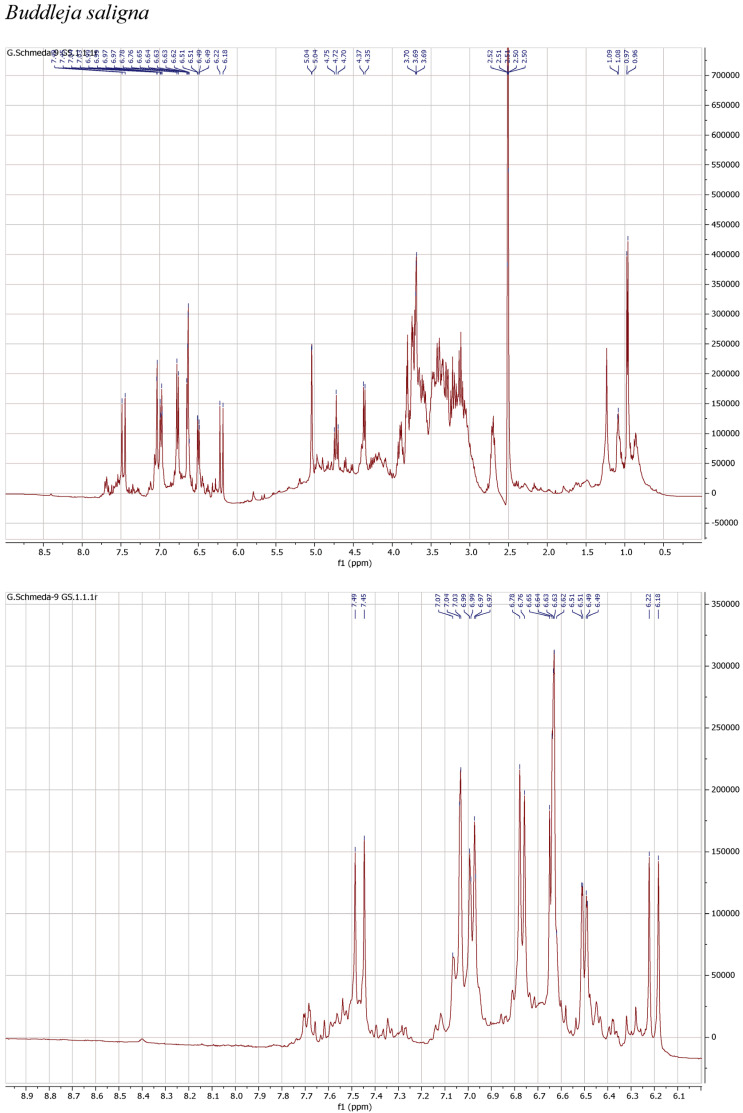

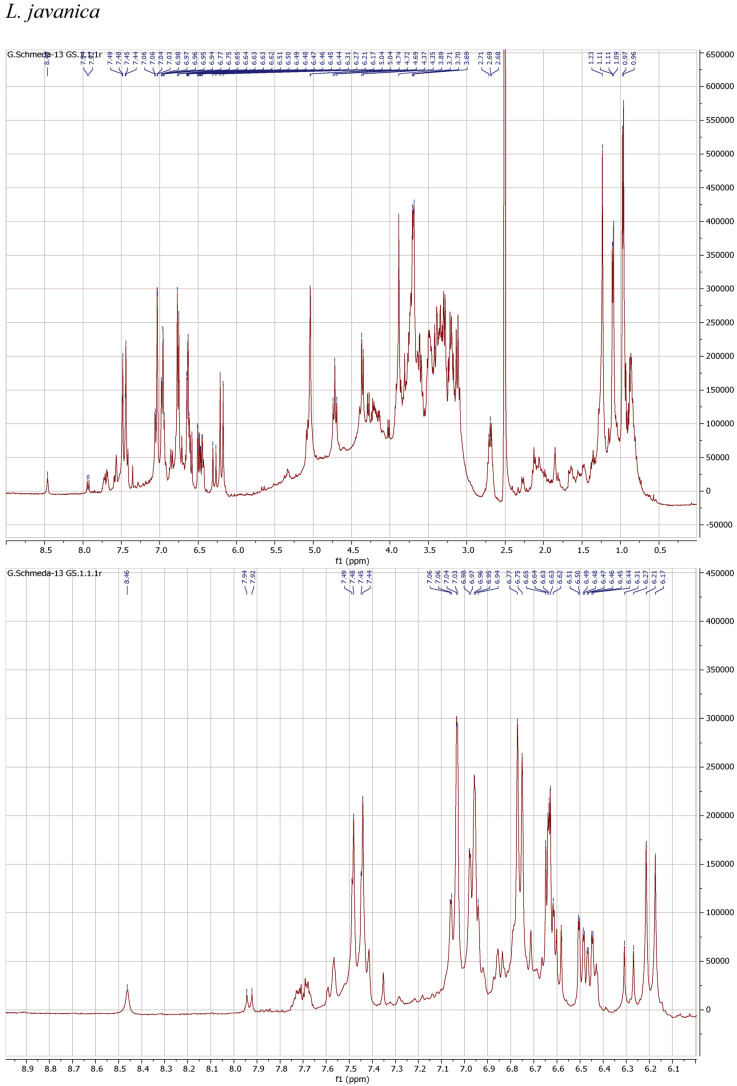

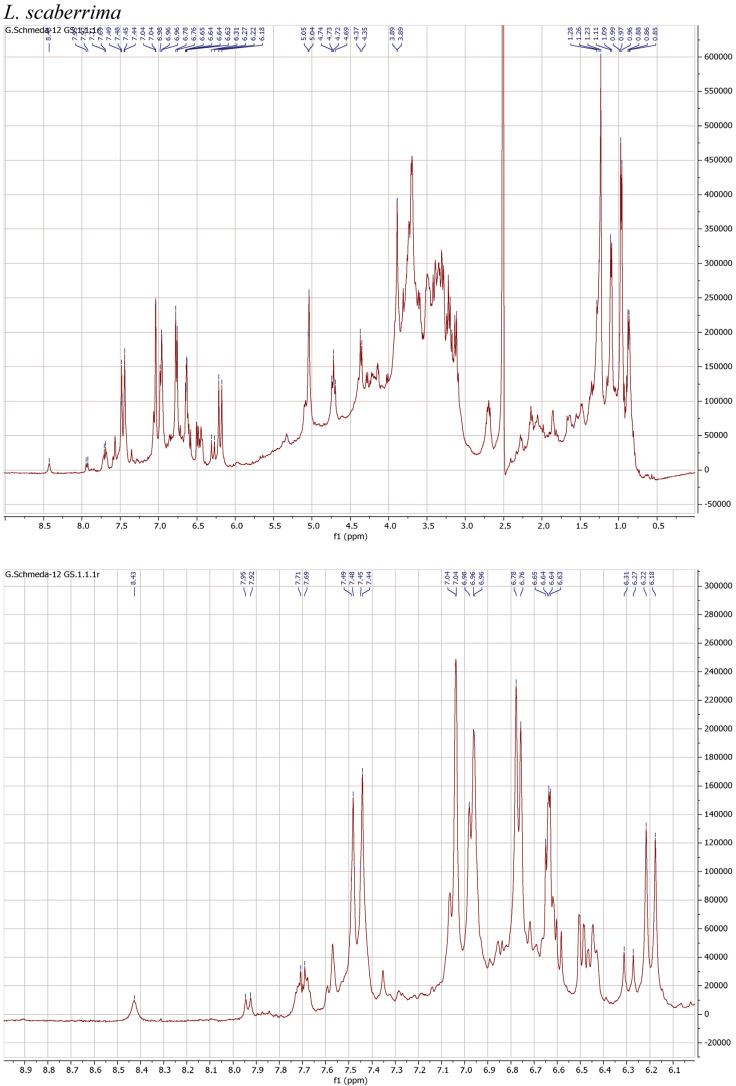

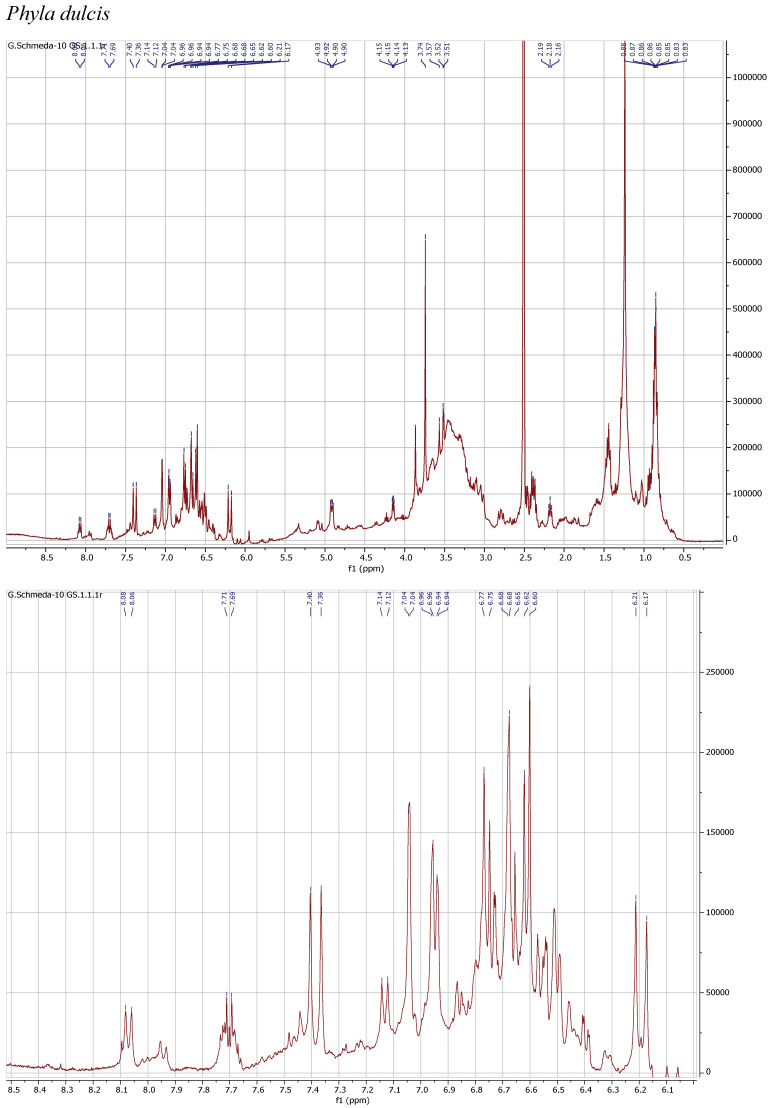
^1^H NMR spectra of the infusions from *Buddleja saligna*, *Lippia javanica*, *L. scaberrima*, and *Phyla dulcis* (400 MHz, DMSO-d6).

^1^H NMR spectra of the herbal teas were measured to obtain a first insight into the teas’ compositions. The spectra and enlarged section of the aromatic signals are presented in Figure 1.

The ^1^H NMR spectrum of the infusion from *B. saligna* shows a main compound with the typical signals for two 1,3,4-trisubstituted aromatic rings belonging to a caffeoyl and a phenylethyl moieties as well as two anomeric sugar H, overlapping multiplets from the sugar protons in the δ 3.1–4.2 range, and a d at δ 0.97, compatible with a deoxy sugar. The signals at δ 7.47 d (*J* = 15.9 Hz) (H-β′), 7.03 d (*J* = 2.2 Hz) (H-2′), 6.98 dd (*J* = 8.3, 2.2 Hz) (H-6′), 6.77 d (*J* = 8.3 Hz) (H-5′), and 6.20 d (*J* = 15.9 Hz) (H-α′) agree with the caffeoyl, while the H at δ 6.64 d (*J* = 7.8 Hz) (H-5), 6.63 d (*J* = 2.1 Hz) (H-2), 6.50 dd (*J* = 7.8, 2.1 Hz) (H-6), 3.90 m (H-α), and 2.70 m (H-β) can be assigned to the phenylethyl aglycone. The signals at δ 5.04 br s and 4.36 d *(J* = 7.3 Hz) from the anomeric H pointed out a rhamnose and a β-hexose, identified as glucose. The d at δ 0.97 (*J* = 6.0 Hz) confirms the rhamnose and the t at 4.72 (*J* = 9.6 Hz) confirms the placement of the caffeic acid ester at the C-4 from the glucose. The NMR data agrees with verbascoside [8].

The teas from *L. javanica* and *L. scaberrima* were very similar and showed a mixture of phenylethanoid glycosides as the main compounds. The main difference with *B. saligna* is the presence of a more complex mixture in *Lippia*, with two products showing the rhamnose methyl at δ 0.97 (main compound) and δ 1.10, respectively, with slight differences in the α-H of the caffeoyl ester (δ 6.20 and 6.29, respectively). The compounds were assigned as verbascoside and isoverbascoside. Both compounds were reported from *L. javanica* [8]. Two additional minor constituents can be deduced from additional d at δ 0.87 and 0.86, supporting the presence of isomers.

The ^1^H NMR spectrum of the PEI from *P. dulcis* shows a main compound with signals at δ 7.38 d (*J* =15.9 Hz; H-7′), 7.04 d (*J* = 2.2 Hz; H-2′), 6.95 dd (*J* = 8.3, 2.2 Hz; H-6′), 6.76 d (*J* = 8.3 Hz; H-5′), 6.61 d (*J* = 7.8 Hz; H-5), 6.68 d (*J* = 2.1 Hz; H-2), 6.50 dd (*J* = 7.8, 2.1 Hz; H-6), and 6.19 d (*J* = 15.9 Hz; H-8′), supporting a caffeoyl acid and an additional phenolic moiety. The dd at δ 4.91 (*J* = 8.7, 3 Hz; H-8) suggests the occurrence of rosmarinic acid, confirmed with a reference sample. Additional signals in the aromatic region and the sugar H support the presence of flavonoid glycosides.

### 3.5. HPLC-DAD Profiles

**Figure 2:**
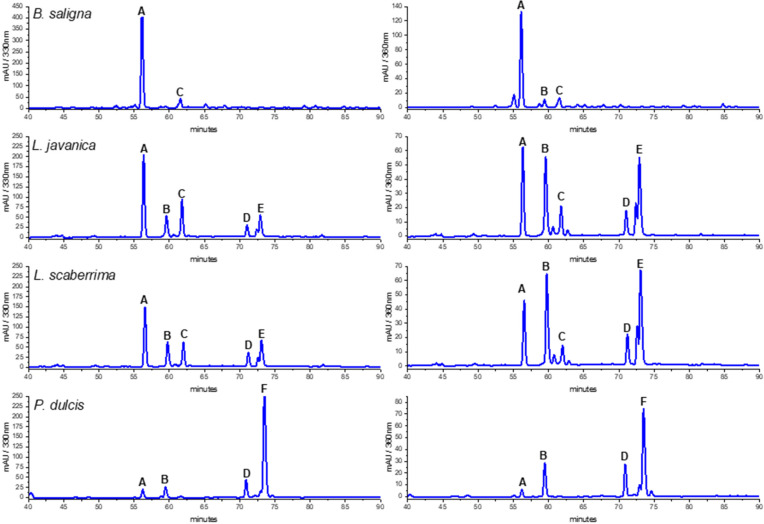
HPLC-DAD profiles of *Buddleja saligna*, *Lippia javanica*, *L. scaberrima*, and *Phyla dulcis* infusions at 330 and 360 nm. Compounds: A: verbascoside; B: Quercetin 3-*O*-glucoside; C: isoverbascoside; D: flavonoid; E: Quercetin derivative; F: Rosmarinic acid.

The HPLC traces of the MeOH and water infusions of the crude drugs were compared to select the better conditions for HPLC-MS/MS analysis. The PEI from the teas provided a better insight into the composition of the product consumed than the MeOH extract, as it was obtained after enrichment of the infusions (Figure 2). The reason for the HPLC-DAD analysis is to give the option for identification and quantification of the main compounds using accessible equipment. Furthermore, the HPLC-DAD fingerprints offer the option for quality control of the crude drug and can be used to compare different samples/populations and changes of composition following crude drug processing/storage.

The HPLC fingerprints of *L. javanica* and *L. scaberrima* were similar, with the same main constituents eluting at R_t_ 56.2 (A), 59.5 (B), 62.0 (C), and 73.1 min (D), respectively. The UV spectra suggest the occurrence of two different groups of compounds, namely phenylethanoid glycosides and flavonoids (Table 4). The main constituent for *L. javanica* and *L. scaberrima* was assigned as verbascoside/acteoside (A) according to the literature [8], while rosmarinic acid (F) is the main product in *P. dulcis*. Verbascoside was the main compound detected in the HPLC trace of *B. saligna*. According to the literature, the UV maxima of verbascoside are λ 326 and 289 nm, while for isoverbascoside they are 322 and 288, respectively [8]. The compounds B and F were identified as quercetin 3-*O*-glucoside and rosmarinic acid, respectively, via a comparison with reference standard compounds (PhytoLab, Vestenbergsgreuth, Germany). The identity of verbascoside was confirmed by NMR analyses.

### 3.6. HPLC-MS/MS Analyses

**Figure 3:**
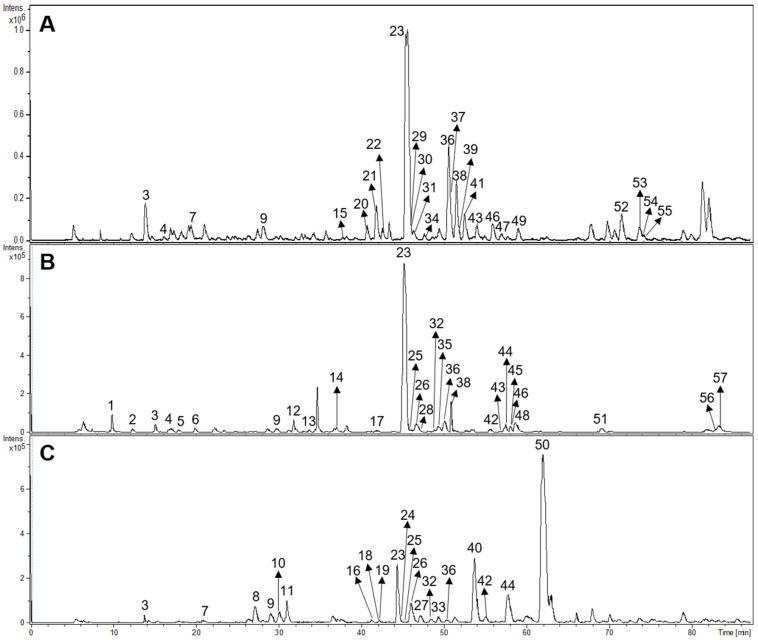
HPLC-ESI-MS/MS chromatograms of *Buddleja saligna* (**A**), *Lippia scaberrima* (**B**), and *Phyla dulcis* (**C**). For the identity of the compounds please see Table 5.

Fifty-seven compounds belonging to different structural groups were tentatively identified from the infusions. They comprised iridoids, flavonoids, phenylpropanoid glycosides, caffeic acid esters, and other compounds. The identification follows from the molecular formula, fragmentation patterns, interpretation of the information, literature, and databases including www.foodb.ca. The HPLC-ESI-MS/MS chromatograms of the samples are shown in Figure 3.

**Figure 4:**
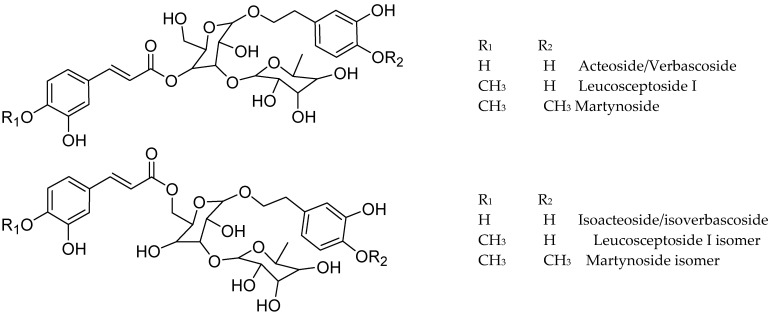

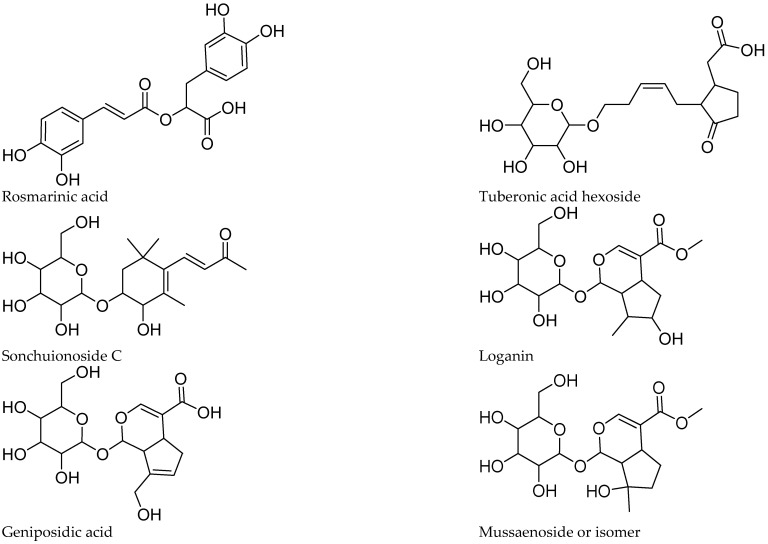
Structure of some compounds identified/tentatively identified from the selected South African teas.

The tentative identification of the tea constituents by HPLC-MS/MS is summarized in Table 5. Some compounds identified in the samples are shown in Figure 4.

#### 3.6.1. Iridoids

Five iridoids were tentatively identified in the extracts, including compounds **1**, **2**, **6**, **12**, and **14**. The proposed identification was supported by the work of [13,14,15] on iridoid glucosides. Compounds **1** and **14** showed a pseudomolecular ion at *m*/*z* 389, compatible with the molecular formula C_16_H_22_O_11_. Both lost a hexose (162 amu), yielding a fragment at *m*/*z* 227, in agreement with theveside and mussaenoside isomers [16]. Considering their previously reported elution order, compound **1** was tentatively identified as theveside and **14** as mussaenoside. Both iridoids were previously informed in *L. alba*, *L. citriodora*, and *L. graveolens* [16,17]. The [M-H]^−^ ion at *m*/*z* 373 of compound **2** and the MS^2^ fragments at *m*/*z* 211, 167, and 123 agree with that reported for geniposidic acid [15]. Compound **12** was detected as [M+HCOOH]^−^ at *m*/*z* 435, yielding the fragments at *m*/*z* 389 and 227, characteristic of loganin [16]. Compound **6** showed [M+HCOOH]^−^ and MS^2^ ions at *m*/*z* 451, 405, and 243, differing in one oxygen from **12** and was assigned as hydroxyloganin. The exact stereochemistry and placement of the hydroxy group remain to be established. 8-epiloganin and lamiide were reported for *L. dulci*, and the first for *L. alba* [16,18], while loganin and its derivatives were informed for *L. graveolens* [19].

#### 3.6.2. Flavonoids

Seven kaempferol derivatives, including a dihexoside (**15**), dirhamnoside pentoside (**19**), hexoside rhamnoside (**22**), three isomeric hexosides (compounds **25**, **31** and **47**) and the glucuronide (**26**) were identified in the teas, showing the characteristic loss of the sugars, leading to the MS^2^ ion of kaempferol at *m*/*z* 285. The position of the sugar residues was theoretically assigned based on the literature [20,21,22]. We proposed that compound **15** was kaempferol 3,7-di-*O*-hexoside, based on the ions at *m*/*z* 447 and 285, and the low formation of the radical ion at *m*/*z* 284, indicating 3-*O* and 7-*O* substitutions, respectively [21,22]. Compound **19** was tentatively assigned as kaempferol 7-*O*-rhamnoside 3-*O*-rhamnoside pentoside because of the intense MS^2^ ion at *m*/*z* 563, consistent with a preferential neutral loss in position 7 and the high formation of the radical ion at *m*/*z* 284, suggesting a 3-*O* substitution [20,22]. Peaks **22**, **26**, and **47** were tentatively identified as 7-*O* substituted derivatives based on the low relative abundance of the radical at *m*/*z* 284, whereas **25** and **31** were assigned as 3-*O* hexosides due to the high formation of this ion radical.

The quercetin glycosides **16**, **21**, **24**, and **30** showed the neutral loss of rutinose, hexose, and rhamnose, as well as one hexose unit, with a base peak in agreement with quercetin; they were assigned as quercetin rutinoside (**16**), hexoside rhamnoside (**21**), and hexosides (compounds **24** and **30**), differing in the identity of the hexose. The position of the sugar residues was inferred to be in 3-*O* due to the detection of the ion at *m*/*z* 271 in all peaks, characteristic of 3-*O*-substituted quercetin derivatives [22].

The compound **28**, with a *m*/*z* of 491.0843 shows the neutral loss of glucuronic acid and further fragments to 315.0519, in agreement with rhamnetin/isorhamnetin, and was identified as rhamnetin/isorhamnetin glucuronide. Eriodictyol hexoside (compound **29**) was detected in the infusion of *B. saligna*, but in neither *L. javanica*, *L. scaberrima* nor *P. dulcis*. (2R) and (2S) eriodictyol 7-*O*-glucopyranoside was reported from several Brazilian *Lippia* species [23]. The compound **32** showed neutral loss of glucuronic acid and a base peak compatible with hesperetin, being assigned as hesperetin glucuronide. The fragmentation of compound **35**, with a *m*/*z* of 507.1181, showed the loss of a hexose followed by two methyl groups, suggesting a dimethoxy myricetin hexoside. The compound was tentatively assigned to syringetin hexoside and the placement of the methoxy groups remains to be established [24].

The mass spectrum of compound **8** shows a UV maximum at 332 nm and fragmentation according to a flavone C-glucoside. The spectrometric data agrees with apigenin 6,8-di-C-hexoside [25]. Compound **44** shows loss of glucuronic acid and a base peak at *m*/*z* 269, in agreement with apigenin 7-*O*-hexuronide [21]. The related compounds **45** and **48** show loss of glucuronic acid, leading to the base peak at *m*/*z* 329 and 299, respectively, and were assigned as the flavones tricin glucuronide and chrysoeriol glucuronide, respectively.

Compound **54**, with a *m*/*z* ion at 491 and molecular formula C_23_H_24_O_12_, shows the neutral loss of hexose and a base peak at *m*/*z* 329, in agreement with quercetin dimethyl hexoside. The aglycones quercetin methyl ether (compound **56**), compatible with rhamnetin/isorhamnetin) and dimethylmyricetin (compound **57**), were tentatively identified by the loss of one or two methyl from the [M-H]^+^ ions.

#### 3.6.3. Phenylpropanoid Glycosides

Phenylpropanoid glycosides were the main compounds in *Lippia* and *Buddleja*, with verbascoside/isoverbascoside occurring in *B. saligna*, *L. javanica*, and *L. scaberrima*.

The compounds **23**, **33**, **36**, and **38** show a molecular formula of C_29_H_35_O_15_ for the [M-H]^-^ ion, and similar fragmentation patterns, in agreement with verbascoside (acteoside) and isoverbascoside (isoacteoside) isomers [8,23,26]. The double bond in the caffeic acid moiety can be *trans* or *cis*, and the placement of the phenolics in the sugar moiety can explain the differences in the R_t_ values in the chromatograms. Compound **23** was identified as verbascoside/acteoside based on the ^1^H NMR analysis of the infusions and **33** was assigned as isoverbascoside/isoacteoside. Compounds **36** and **38** were tentatively assigned as verbascoside/ isoverbascoside isomers 1 and 2, respectively. According to [26], the elution sequence for the isomeric compounds forsythioside A, acteoside, *cis*-acteoside, and isoacteoside was an R_t_ of 26.953, 27.497, 28.200, and 30.331 min, respectively. Acteoside, isoacteoside, martynoside, and diacetylmartinoside were reported from *L. dulcis* cultivated in Japan [18]. Verbascoside (Acteoside) (compound **23**) was identified in *Lippia* and *Buddleja* as a main compound, in agreement with literature. The related compound **3** shows a molecular formula of [M-H]^+^ C_20_H_29_O_12_, and differs from verbascoside by a caffeoyl acid moiety, being assigned as a decaffeoyl verbascoside isomer.

Compound **34** shows typical fragmentation for a phenylpropanoid glycoside and differs from verbascoside/isoverbascoside by one deoxysugar unit. The compound agrees with calceolarioside B (desrhamnosyl isoacteoside) [27].

The compounds **37**, **42**, and **46** showed a molecular formula differing from that of acteoside/isoacteoside by one methoxy group instead a hydroxy in the aromatic moiety. Fragmentation of the [M-H]^-^ ion at *m*/*z* 637 led to the base peak at *m*/*z* 175, in agreement with Leucosceptoside A [16,28]. Compounds **42** and **46** were assigned as Leucosceptoside A isomer 1 and isomer 2, respectively. The fragmentation of compound **37** shows differences in the placement of the OCH_3_ in the aromatic moieties. While, in **42** and **46** the methoxy is placed in the phenylpropanoid moiety (ferulic acid), in compound **37** the phenylpropanoid agrees better with coumaric acid (*m*/*z* 163 and 145), and the methoxy group should be in the phenylethanoid moiety. A related compound was reported from *Forsythia suspensa* [29]. Compound **41** showed a molecular formula and fragmentation compatible with a methoxycaffeic acid dihexoside bearing an additional methoxyphenyl ethanoid moiety and was assigned as hydroxy hemiphroside A [30].

Compounds **51** and **53** showed a [M-H]^-^ ion at *m*/*z* 651 and a molecular formula of C_31_H_39_O_15_, differing from verbascoside/isoverbascoside by the presence of methoxy instead of hydroxy in the phenolic moiety of the glycoside, and were assigned as martynoside [28] isomer 1 and 2, respectively. Leucosceptoside A and martynoside are the monomethyl ether of acteoside/isoacteoside and the dimethylether of acteoside/isoacteoside, respectively. Compound **55**, with a molecular formula of C_35_H_45_O_20_, lost one caffeoyl moiety (162 amu) and yielded MS^2^ fragments at *m*/*z* 623, 461, and 161, in agreement with acteoside/verbascoside [26]. Thus, **55** was tentatively assigned as echinacoside [31].

Compounds **13** and **17** differ from acteoside/isoacteoside by the presence of one additional hydroxy group (compound **13**) or methoxy group (compound **17**) and were tentatively identified as hydroxy acteoside and methoxy acteoside, respectively. Compound **43**, with a [M-H]^-^ ion at *m*/*z* 607 and a molecular formula of C_29_H_35_O_14_, differs from verbascoside/isoverbascoside by one oxygen, and the fragmentation pattern agrees with that reported for desoxyacteoside [32].

The mass spectrum of compound **39** shows a molecular formula of C_30_H_37_O_16_, and fragments to the base ion at *m*/*z* 161, supporting a caffeic acid derivative. The fragmentation and molecular formula are compatible with campneoside [32]. The related compound **49** shows the loss of 180 amu, leading to *m*/*z* 487 and a base peak at *m*/*z* 193, in agreement with scroside B. The molecular formula and fragmentation pattern agree with data reported by [30]. Compound **4** was assigned as Cistanoside F by its molecular formula and fragmentation, in agreement with the literature [33].

#### 3.6.4. Caffeic Acid Esters

Caffeic acid (compound **11**) was identified in *P. dulcis* by the UV spectrum and molecular formula and occurs as the hexoside (compound **7**) in *P. dulcis* and *B. saligna*. Compound **20** shows a [M-H]^+^ ion at *m*/*z* 667 and a molecular formula of C_30_H_35_O_17_. The fragmentation shows the neutral loss of a caffeoyl moiety (162 amu) and the ions at *m*/*z* 505, 170, and 161 (base peak), in agreement with Hebitol II [26].

The caffeic acid ester rosmarinic acid (compound **50**) occurs in *P. dulcis* and was identified by its molecular formula, fragmentation pattern, and comparison with a reference standard compound. Compounds **18** and **27** show an *m*/*z* of 521 and the [M-H]^+^ ion with a molecular formula of C_24_H_25_O_13_, with a base peak at *m*/*z* 161, and in agreement with rosmarinic acid hexosides. The glycosides were assigned as rosmarinic acid hexoside 1 (**18**) and hexoside 2 (**27**), respectively. Compound **40** showed a [M-H]^+^ ion at *m*/*z* 719 and a molecular formula of C_36_H_31_O_16_, suggesting four phenylpropanoid units and fragments with a base peak at *m*/*z* 161. The fragmentation and molecular formula suggest the occurrence of a sagerinic acid isomer. The compound was tentatively assigned as sagerinic acid, in agreement with [34,35].

#### 3.6.5. Other Compounds

Tuberonic acid hexoside **9** was identified by its molecular formula and fragmentation, according with previous studies on *B. officinalis* flowers [26]. In our sample, compound **10** with the pseudomolecular ion [M+HCOOH]^+^ at *m*/*z* 431.1920 and a molecular formula of C_19_H_29_O_8_ shows the neutral loss of hexose, leading to the base peak at *m*/*z* 223, which further fragments to *m*/*z* 153, in agreement with sonchuionoside C [36].

Compound **5**, with a molecular formula of [M-H]^+^ C_15_H_19_O_10_, shows the neutral loss of hexose, leading to the base peak at *m*/*z* 197, in agreement with syringic acid. The compound was identified as syringic acid hexoside. Compound **52** showed the neutral loss of a pentose, leading to a base peak at *m*/*z* 289, in agreement with octen-3-yl hexoside and was assigned as the acyl carbohydrate 1-octen-3-yl hexoside pentoside. The occurrence of the different compounds in the infusions of the selected South African teas is summarized in Table 5.

## 4. Discussion

The infusions and MeOH extracts of selected herbal teas from South Africa were assessed for the inhibition of enzymes associated with metabolic syndrome. All infusions and extracts showed good inhibition of the α-glucosidase. *Buddleja saligna* is traditionally used for diabetes in South Africa. Different leaf extracts of the plant were previously assayed for α-glucosidase inhibition [37]. According to [37], the hexane extract showed the best α-glucosidase inhibition (IC_50_ = 260 μg/mL). However, our results using the infusion and a polar methanol extract show a lower IC_50_ (higher activity) due to a different chemical composition, resembling that of traditional preparations and herbal teas.

The inhibitory effect of the MeOH extracts towards the α-glucosidase was 0.47–0.50 µg/mL while the activity of the PEI was higher for *B. saligna* and *P. dulcis* (0.21 and 0.13 µg/mL, respectively). The MeOH extracts of the crude drugs showed a TP content ranging from 9.43 to 11.66 g/100 g extract, while the PEI of *B. saligna* and *P. dulcis* contains 29.29 and 17.69 g GAE/100 g extract, respectively. The increase in activity can be explained by higher phenolics and by the identity of the compounds occurring in the samples. The α-glucosidase inhibition by the PEI of *L. javanica* and *L. scaberrima*, with a higher TP than the MeOH extract, however, did not show relevant changes, with IC_50_ values of 0.43 and 0.84 µg/mL, respectively. Rosmarinic acid and verbascoside were the main constituents in the *P. dulcis* and *B. saligna* extracts, respectively.

The enzymes α-glucosidase, α-amylase, and pancreatic lipase have different substrates and specificity. The enzyme α-glucosidase is a hydrolase that releases α-glucose from carbohydrates. The enzyme acts by hydrolyzing terminal glycosidic bonds, releasing α-glucose from the nonreducing end of the substrate chain. α-Amylase, a starch hydrolase, is the main digestive enzyme in saliva. It begins the digestion of starch hydrolyzing α-1,4 glycosidic linkages and releases smaller molecules such as disaccharides or trisaccharides. Pancreatic lipase hydrolyzes the ester linkages of triglycerides releasing fatty acids and glycerol. The enzyme is relevant for lipid absorption and digestion of cholesterol and fatty acid esters as well as lipid-soluble vitamins. As the selected enzymes have different structures and biochemical targets, the effect of the plant constituents will be different for the three enzymes. Selectivity is a desired characteristic for a possible application in pharmaceutical food science. A recent review on natural compounds affecting glucose and lipid metabolism shows the potential of this approach [38].

The composition of the teas was determined by HPLC-DAD-MS/MS and ^1^H NMR. Chemical profiles using HPLC-DAD allow for the identification of the main compounds in the infusions and methanol extracts. The six main phenolics, including the differential marker rosmarinic acid for *P. dulcis*, the phenylpropanoids verbascoside and isoverbascoside occurring in different ratios in *B. saligna*, *L. javanica*, and *L. scaberrima*, and the flavonoids, can be used for quality controls of the crude drugs.

The HPLC-DAD fingerprints strategy, using reference compounds, is a suitable method to monitor changes in composition related to different populations, select the best harvesting time, and set the basis for further studies on changes related to environmental responses and processing for tea production. Using the more sensible HPLC-MS/MS analyses, 26 compounds were identified in the infusion from *B. saligna*, 28 from *L. scaberrima*, and 21 from *P. dulcis*. A few of them (decaffeoylverbascoside, verbascoside, isoverbascoside, and tuberonic acid hexoside) were found in all the samples. In addition, 10 compounds occur in two of the species. Most of the compounds occurring in low amounts were species-specific, accounting for seventeen, fifteen, and eleven metabolites in *B. saligna*, *L. scaberrima*, and *P. dulcis*, respectively. Most of the compounds reported in this work are described for the first time from the selected South African crude drugs and the few products previously identified in the plants are indicated with an * in Table 5.

The phenylpropanoid glycosides verbascoside and isoverbascoside have been reported as the main phenolics from the aerial parts of *L. javanica* [8]. Both compounds were isolated from the defatted MeOH extract of the plant and were fully identified by spectroscopic means and comparison with standards. Verbascoside and isoverbascoside were also quantified in MeOH:H_2_O 80:20 extracts from *Lippia* species, including *L. scaberrima* [8]. Verbascoside, also known as acteoside, inhibits the growth of tumor cells [39], reduces sugar absorption, protects pancreatic β-cells against endoplasmic reticulum (ER) stress [40] and, acts as a hepatoprotective, antioxidant, and anti-inflammatory, among other functions [41]. Rosmarinic acid is a well-known antioxidant with anti-inflammatory effects [42], which shows inhibition towards several enzymes and strong therapeutic potential [43,44]. Related studies have been performed with herbal teas from the Anatolian peninsula [45], China [46], and South Africa [47].

Another study on South African crude drugs investigated in this work includes the potential of an ethanol extract from *B. saligna* in sunscreen formulations [48]. However, the phytochemical information in this study was obtained by GC-MS analyses of an ethanol extract and the polar constituents occurring in the extract were not identified due to the analytical method. Polar *Buddleja* constituents were described by [26] in *B. officinalis* flowers and polyphenols from *B. globosa* leaves were reported by [32]. The effect of *B. saligna* on the angiotensin converting enzyme and the mechanism involved in lipid metabolism were described [49]. Some triterpenes were isolated and identified from leaves of *B. saligna* [50].

The in vitro antimycobacterial activity of *Lippia scaberrima* was reported by [9], along with the phenolic content, antibacterial, and antioxidant in vitro from South African *Lippia* infusions [51]. A dereplication study on Brazilian *Lippia* species showed that verbascoside occurs in most of the samples, while isoverbascoside was detected in six out of ten species [23]. A recent work on *P. dulcis*, using the synonym *Lippia dulcis* was published [52] with Brazilian material. The authors identified verbascoside by co-chromatography with a standard sample and worked on different extracts obtained with methanol or ethanol. Our extract of *P. dulcis* shows a different composition, with rosmarinic acid as a main constituent; this supports the importance of verifying the botanical identity and chemical composition of herbal teas in the production place. Neither hernandulcin nor lippidulcine derivatives were detected in the South African grown *P. dulcis*. In a study on plant material from Japan, the isolation and identification of (+)hernandulcin, (−)epihernandulcin, lippidulcine A, and epilippidulcine was reported [18].

Methanol extracts and infusions of four different South African herbal teas were compared for composition, antioxidant capacity, and inhibition of enzymes related to metabolic syndrome (α-glucosidase, α-amylase, and pancreatic lipase). Three of the species belong to the Verbenaceae family and one of them to the Scrophulariaceae. The four herbal teas inhibited the enzyme α-glucosidase with a better effect for the phenolic-enriched infusions (PEI) of *Phyla dulcis* and *Buddleja saligna* (IC_50_ values of 0.13 and 0.21 µg/mL, respectively). The PEI was mildly active towards α-amylase at 100 µg/mL, suggesting some potential for inhibiting these two enzymes, related to sugar absorption. The metabolites occurring in the teas, including rosmarinic acid, verbascoside, and isoverbascoside among others, might explain, at least in part, the effect of the selected South African herbal teas as inhibitors of the α-glucosidase and α-amylase. Verbascoside was reported as an inhibitor of α-glucosidase in Brazilian *Lippia dulcis*, a botanical synonym of *P. dulcis*. The molecular docking and enzyme inhibition of the compound described in [52] confirm its effect on α-glucosidase and help explain the effect of the infusions as strong glucosidase inhibitors in the South African samples. Rosmarinic acid has been reported as an α-glucosidase inhibitor and was isolated from different plant sources, including *Rosmarinus officinale*, *Perilla* leaves [53], and *Rabdosia serra* [54]. Other reported effects of the compounds included antioxidant and antiallergic properties [53], and tyrosinase inhibition [54]. In an in silico reverse docking investigation, rosmarinic acid and flavonol quercetin were identified as inhibitors of the enzymes α-glucosidase and pancreatic α-amylase, and as inhibitors of lipid accumulation in hepatic cells [55]. Taking all this information together, the occurrence of rosmarinic acid as a major compound in *P. dulcis* supports the notion of herbal tea as a digestive.

The comparison of the samples by HPLC-DAD shows that *B. saligna*, *L. javanica*, and *L. scaberrima* are phenylpropanoid glycosides-containing herbal teas while *P. dulcis* contains rosmarinic acid as a main bioactive. Our work presents an overview of the chemical constituents of the infusions that can be used for the characterization and quality control of herbal teas. The chemical fingerprints observed in HPLD-DAD allow a clear differentiation among *B. saligna*, *P.dulcis*, and the two *Lippia* species (*L. javanica* and *L. scaberrima*). Further work is needed to find additional chemical and/or molecular markers to differentiate *L. javanica* and *L. scaberrima*. The fingerprints with the main phenolics from the crude drugs can be used to start new studies on the variability of the constituents and enzyme inhibition according to the production places, agricultural practices, and response to environmental stress.

## Figures and Tables

**Table 1:** Extraction yield from the South African herbal teas using MeOH and PEI of infusion.

Scientific Name	Plant Family and Plant Part	Trade Name	% Yield MeOH Extract	% Yield PEI of Infusion
*Buddleja saligna* Willd.	Scrophulariaceae, L	Gancair	23.11	5.01
*Lippia javanica* (Burm.f.) Spreng	Verbenaceae, L	Zinibar	6.82	4.91
*Lippia scaberrima* Sond.	Verbenaceae, L	Mosukujane	6.16	4.07
*Phyla dulcis* (Trevir.) Moldenke	Verbenaceae, L	Haw Haw	12.83	3.95

L: leaves.

**Table 2:** Total phenolic (TP), total flavonoid (TF), total proanthocyanidin (TPA) content, and antioxidant activity (DPPH, FRAP, TEAC, ORAC) of infusions and methanol extracts from selected South African herbal teas.

Plant Species and Extract Type	TP (g GAE/100 g Extract)	TF (g CE/100 g Extract)	TPA (g CE/100 g Extract	DPPH (SC_50_, µg/mL)	FRAP (µmol TE/g Extract)	TEAC (µM TE/g Extract)	ORAC (µmol TE/g Extract)
MeOH extract							
*Buddleja saligna*	9.71 ± 0.11 ^a^	0.79 ± 0.03 ^a^	not detected	23.62 ± 0.39 ^a^	221.31 ± 6.37 ^a^	766.92 ± 30.71 ^a^	197.14 ± 16.76 ^a^
*Lippia javanica*	11.66 ± 0.07 ^b^	0.97 ± 0.05 ^a,b^	not detected	18.28 ± 0.48 ^b^	267.28 ± 9.57 ^b^	1091.43 ± 45.50 ^b^	182.55 ± 6.20 ^a^
*Lippia scaberrima*	10.79 ± 0.12 ^c^	0.99 ± 0.04 ^b^	not detected	22.42 ± 0.80 ^a^	260.92 ± 16.21 ^b^	806.97 ± 32.50 ^a^	271.17 ± 10.15 ^b^
*Phyla dulcis*	9.43 ± 0.10 ^d^	2.86± 0.18 ^c^	0.48 ± 0.03	26.70 ± 1.01 ^c^	337.31 ± 14.44 ^c^	1019.69 ± 40.03 ^b^	192.33 ± 13.58 ^a^
PEI							
*Buddleja saligna*	29.29 ± 0.61 ^a^	13.82 ± 0.39 ^a^	not detected	5.16 ± 0.10 ^a^	1277.18 ± 60.17 ^a^	2214.17 ± 114.90 ^a^	909.49 ± 78.49 ^a^
*Lippia javanica*	17.95 ± 0.03 ^b^	12.13 ± 0.58 ^b,d^	not detected	5.97 ± 0.05 ^b^	1028.90 ± 24.10 ^b^	1609.18 ± 83.01 ^b^	518.51 ± 17.58 ^b^
*Lippia scaberrima*	23.84 ± 0.41 ^c^	22.94 ± 0.04 ^c^	0.31 ± 0.01	7.00 ± 0.26 ^c^	977.88 ± 55.62 ^b^	1528.75 ± 76.69 ^b^	423.92 ± 28.62 ^b^
*Phyla dulcis*	17.69 ± 0.17 ^b^	13.01 ± 0.15 ^a,d^	2.81 ± 0.16	5.77 ± 0.20 ^b^	1805.56 ± 38.90 ^c^	2025.03 ± 104.81 ^a^	1037.81 ± 12.48 ^c^
Quercetin ^#^	-	-	-	8.05 ± 0.41	1090.23 ± 17.45	8180.66 ± 20.88	23374.06 ± 897.39

PEI: phenolic-enriched infusion; DPPH: 2,2-diphenyl-1-picrylhydrazyl radical; FRAP: ferric reducing antioxidant power; TEAC: Trolox equivalents antioxidant capacity; ORAC: oxygen radical antioxidant capacity; GAE: gallic acid equivalents; CE: catechin equivalents; SC_50_: extract concentration scavenging 50% of the DPPH radical; TE: Trolox equivalents; -: not determined; #: reference compound. Results are the mean values ± SD of three independent experiments. Different superscript letters (a–d) in the same column show significant differences within each extraction method, according to Tukey’s test (*p* < 0.05).

**Table 3:** Inhibitory activity of the PEIs and MeOH extracts from South African herbal teas towards α-glucosidase, α-amylase, and pancreatic lipase.

Sample	α-Glucosidase (IC_50_, µg/mL)	α-Amylase (% at 100 µg/mL or IC_50_, µg/mL)	Lipase (% at 50 µg/mL or IC_50_, µg/mL)
MeOH			
*Buddleja saligna*	0.47 ± 0.03 ^a^	inactive	inactive
*Lippia javanica*	0.47 ± 0.02 ^a^	inactive	inactive
*Lippia scaberrima*	0.49 ± 0.02 ^a^	0.47 ± 0.00%	inactive
*Phyla dulcis*	0.50 ± 0.03 ^a^	inactive	inactive
PEI			
*Buddleja saligna*	0.21 ± 0.02 ^a^	23.14 ± 1.96%	7.25 ± 0.38%
*Lippia javanica*	0.43 ± 0.01 ^b^	34.27 ± 2.04	6.87 ± 0.65%
*Lippia scaberrima*	0.84 ± 0.13 ^c^	28.61 ± 1.63%	inactive
*Phyla dulcis*	0.13 ± 0.06 ^a^	23.03 ± 2.01%	inactive
Acarbose ^#^	137.73 ± 1.31	28.48 ± 0.29	-
Orlistat ^#^	-	-	0.04 ± 0.00

PEI: phenolic-enriched infusion; IC_50_: concentration that inhibits 50% of the enzyme activity; %: percentage inhibition in the enzymatic activity; #: reference compounds. Results are the mean values ± SD of three independent experiments. Different superscript letters (a–c) in the same column show significant differences within extraction methods, according to Tukey’s test (*p* < 0.05).

**Table 4:** Main compounds detected in the HPLC-DAD traces of *Buddleja saligna*, *Lippia javanica*. *L*. *scaberrima*, and *Phyla dulcis*.

Peak	Rt (min)	UV Maxima (nm)	Identification	Sample
A	56.2	330, 288sh, 245, 219sh	Verbascoside	*B. saligna*, *L. javanica*, *L. scaberrima*, *P. dulcis*
B	59.5	348, 268sh, 254	Quercetin-3-*O*-glucoside	*L. javanica*, *L. scaberrima*, *P. dulcis*
C	62.0	326, 288sh, 245sh	Isoverbascoside	*B. saligna*, *L. javanica*, *L. scaberrima*
D	71.0	337, 267	Flavonoid	*L. javanica*, *L. scaberrima*, *P. dulcis*
E	73.1	346, 268sh, 252	Quercetin derivative	*L. javanica*, *L. scaberrima*
F	73.5	328, 288sh, 244sh	Rosmarinic acid	*P. dulcis*

Detection: UV, 330 and 360 nm.

**Table 5:** Compounds tentatively identified in the MeOH extracts of Gancair (*Buddleja saligna*), Mosukujane (*Lippia scaberrima*), and Haw Haw (*Phyla dulcis*) through LC-MS in negative ion mode, and occurrence of the compounds in the crude drugs.

Peak	Rt (Min)	UV Max	[M-H]^−^ Measured	Molecular Formula	[M-H]^−^ Theoretical	Error (ppm)	MS/MS	Tentative Identification	*B. saligna* (Gancair)	*L. scaberrima* (Mosukujane)	*P. dulcis* (Haw Haw)
1	9.9		389.1086	C_16_H_21_O_11_	389.1089	0.77	389.1072(100), 227.0588(16), 209.0441(40), 191.0352 (55)	Theveside		X	
2	12.5		373.1132	C_16_H_21_O_10_	373.1140	2.14	211.0643 (84), 167.0732 (54), 123.0457 (100)	Geniposidic acid		X	
3	15.3		461.1659	C_20_H_29_O_12_	461.1664	1.08	461.1678 (100), 315.1027 (4), 113.0244 (21)	Decaffeoylverbascoside	X	X	X *
4	17.1		487.1475	C_21_H_27_O_13_	487.1457	−3.69	179.0366 (100), 161.0253 (27), 135.0451 (27)	Cistanoside F	X	X	
5	18.1		359.0987	C_15_H_19_O_10_	359.0984	−0.83	197.0471 (100)	Syringic acid hexoside		X	
6	20.1		451.1473	C_18_H_27_O_13_	451.1457	−3.54	405.1410 (100), 243.0877 (80)	Hydroxyloganin isomer [M+HCOOH]^−^		X	
7	21.1		341.0872	C_15_H_17_O_9_	341.0878	1.75	179.0355(100)	Caffeoyl hexoside	X		X
8	27.2	332	593.1503	C_27_H_29_O_15_	593.1512	1.51	593.1473 (100), 383.0749 (61), 353.0655 (98)	Apigenin 6,8-di-C-hexoside			X
9	29.0		387.1652	C_18_H_27_O_9_	387.1660	2.06	387.1657 (100), 207.1038 (13)	Tuberonic acid hexoside	X	X	X
10	30.1		431.1920	C_20_H_31_O_10_C_19_H_29_O_8_	431.1922	0.46	385.1851 (100) [M-H]^+^ (C_19_H_29_O_8_), 223.1339 (50), 153.0922 (42)	Sonchuionoside C [M+HCOOH]^−^			X
11	31.1	323	179.0359	C_9_H_7_O_4_	179.0350	−5.02	135.0456 (100)	Caffeic acid			X
12	31.9		435.1523	C_18_H_27_O_12_	435.1508	−3.44	389.1452 (16) [M-H]^+^ (C_17_H_25_O_10_), 227.0931 (100), 101.0268 (37)	Loganin [M+HCOOH]^-^		X	
13	33.7		639.1953	C_29_H_35_O_16_	639.1930	−3.59	161.0250 (100)	Hydroxy acteoside		X	
14	37.0		389.1089	C_16_H_21_O_11_	389.1089	0.20	227.0905 (24), 161.0101(100)	Mussaenoside		X	
15	39.1		609.1417	C_27_H_29_O_16_	609.1461	7.22	447.0837 (56), 285.0387 (100), 284.0291(14)	Kaempferol 3,7-di-*O*-hexoside	X		
16	41.3		609.1440	C_27_H_29_O_16_	609.1461	3.45	300.0289 (100), 301.0331(35), 271.0235 (38), 179.0012 (16)	Rutin (Quercetin 3-*O*-rhamnoside glucoside) (rutinoside)			X
17	41.7–42.5		653.2035	C_30_H_37_O_16_	653.2087	7.96	416.8155 (11), 161.0248 (100)	Methoxy acteoside		X	
18	42.2		521.1292	C_24_H_25_O_13_	521.1301	1.72	359.0749 (35), 161.0252 (100)	Rosmarinic acid hexoside 1			X
19	42.3		709.1979	C_32_H_37_O_18_	709.1985	0.85	709.1967(100), 563.1320 (64), 430.0908 (28), 285.0407(30), 284.0311 (75), 255.0283(10)	Kaempferol 7-*O*-rhamnoside 3-*O*-rhamnoside pentoside			X
20	42.2	326	667.1847	C_30_H_35_O_17_	667.1879	4.49	505.1532(44), 179.0353(37), 161.0246(100)	Caffeoyl hebitol II	X		
21	43.2	349	609.1437	C_27_H_29_O_16_	609.1461	3.93	300.0273 (100), 301.0335(40), 271.0237(11)	Quercetin 3-*O*-hexoside rhamnoside	X		
22	44.0	347	593.1480	C_27_H_29_O_15_	593.1511	5.22	285.0394 (100), 284.0316(10)	Kaempferol 7-*O*-hexoside rhamnoside	X		
23	44.2–45.9	329	623.1998	C_29_H_35_O_15_	623.1981	−2.72	315.1091 (11), 161.0260 (100)	Verbascoside/Acteoside	X	X *	X *
24	44.8		463.0875	C_21_H_19_O_12_	463.0882	1.51	301.0397(73), 300.0259 (100), 271.0252 (99)	Quercetin 3-*O*-hexoside 1			X
25	45.2–46.0		447.0943	C_21_H_19_O_11_	447.0933	−2.23	285.0408 (100), 284.0305(68)	Kaempferol 3-*O*-hexoside 1		X	X
26	45.9–46.8	347	461.0724	C_21_H_17_O_12_	461.0726	0.43	285.0414 (100), 284.0360(2)	Kaempferol 7-*O*-glucuronide		X	X
27	46.9–48.0		521.1311	C_24_H_25_O_13_	521.1301	−1.91	521.1288 (29), 359.0795 (53), 323.0780 (27), 179.0360 (34), 161.0251 (100)	Rosmarinic acid hexoside 2			X
28	47.2		491.0843	C_22_H_19_O_13_	491.0831	−2.44	491.0851 (51), 315.0519 (84), 300.0292 (100), 204.0475 (9), 147.0666 (11)	Rhamnetin/isorhamnetin glucuronide		X	
29	47.3		449.1102	C_21_H_21_O_11_	449.1089	−2.89	287.0596 (100), 135.0465 (46)	Eridictyol hexoside	X		
30	47.3		463.0889	C_21_H_19_O_12_	463.0882	−1.51	301.0350 (50), 300.0273 (99), 271.0259 (100)	Quercetin 3-*O*-hexoside 2	X		
31	47.7		447.0928	C_21_H_19_O_11_	447.0933	1.11	284.0350 (100), 285.0397(73)	Kaempferol 3-*O*-hexoside 2	X		
32	48.5		477.1053	C_22_H_21_O_12_	477.1039	−2.93	300.0926 (100)	Hesperetin glucuronide		X	X
33	49.3		623.1999	C_29_H_35_O_15_	623.1981	−2.89	315.1048 (18), 161.0261 (100)	Isoverbascoside/isoacteoside			X *
34	49.1		477.1387	C_23_H_25_O_11_	477.1402	3.14	179.0356 (7), 161.0258 (100)	Calceolarioside B	X		
35	49.3		507.1172	C_23_H_23_O_13_	507.1144	−5.52	345.0642 (22), 329.0340 (100), 314.01112 (42)	Syringetin hexoside		X	
36	49.9–51.8		623.1979	C_29_H_35_O_15_	623.1981	0.32	461.1670 (1), 315.1099 (6), 161.0253 (100)	Verbascoside/Isoverbascoside isomer 1	X	X *	X *
37	52.6		637.2135	C_30_H_37_O_15_	637.2137	−0.31	637.2138(100), 163.0428 (79), 145.0309 (37)	*Leucosceptoside A related derivative*	X		
38	52.8		623.1962	C_29_H_35_O_15_	623.1981	3.04	461.1658 (5), 269.0461 (8), 161.0256 (100)	Verbascoside/Isoverbascoside isomer 2	X	X	
39	52.8		653.2068	C_30_H_37_O_16_	653.2087	2.90	429.1461 (14), 161.0281 (100)	Campneoside I	X		
40	53.7–54.2	282	719.1635	C_36_H_31_O_16_	719.1618	−2.36	719.1631 (40), 359.0773 (14), 197.0462 (41), 161.0247 (100)	Sagerinic acid isomer			X
41	53.9	326	683.2199	C_31_H_39_O_17_	683.2192	−1.02	193.0518 (100)	Hydroxy hemiphroside A	X		
42	55.8		637.2182	C_30_H_37_O_15_	637.2137	−7.06	461.1648 (7), 315.1113 (8), 175.0418 (100)	Leucosceptoside A isomer 1		X	X
43	56.7–56.9	314	607.2047	C_29_H_35_O_14_	607.2032	−2.47	315.1052 (5), 163.0420 (15), 145.0311 (100)	Desoxyacteoside	X	X	
44	57.5	336	445.0775	C_21_H_17_O_11_	445.0776	0.22	445.0748 (7), 270.0502 (16), 269.0453 (100)	Apigenin 7-*O*-hexuronide		X	X
45	57.6–58.1		505.1015	C_23_H_21_O_13_	505.0988	−5.34	329.0663 (100), 314.0455 (88), 299.0208 (66), 148.0556 (23)	Tricin glucuronide		X	
46	57.8–58.3		637.2147	C_30_H_37_O_15_	637.2137	−1.56	315.0494 (19), 175.0419 (100)	Leucosceptoside A isomer 2	X	X	
47	58.5		447.0933	C_21_H_19_O_11_	447.0933	0.02	285.0420 (100), 284.0318 (1)	Kaempferol 7-*O*-hexoside	X		
48	58.6	349	475.0914	C_22_H_19_O_12_	475.0882	−6.73	299.0583 (62), 284.0349 (100), 113.0245 (9)	Chrysoeriol glucuronide		X	
49	60.6		667.2247	C_31_H_39_O_16_	667.2243	−0.59	487.1257 (17), 193.0510 (100)	Scroside B	X		
50	61.8–63.0	330	359.0781	C_18_H_15_O_8_	359.0772	−2.50	197.0466 (54), 161.0255 (100), 133.0301 (57)	Rosmarinic acid			X
51	69.1		651.2319	C_31_H_39_O_15_	651.2294	−3.83	502.9688 (10), 329.1591 (14), 175.0413 (100)	Martynoside		X	
52	72.6		421.2054	C_19_H_33_O_10_	421.2079	5.93	289.1652 (100), 130.9705 (93)	1-Octen-3-yl hexoside pentoside	X		
53	74.8		651.2298	C_31_H_39_O_15_	651.2294	−0.61	476.8643 (19), 442.8783 (22), 175.0407 (100)	Martynoside isomer 1	X		
54	75.2		491.1174	C_23_H_23_O_12_	491.1195	4.27	329.0658 (100), 314.0437 (33), 299.0207 (77)	Quercetin dimethyl hexoside	X		
55	75.3		785.2282	C_38_H_41_O_18_	785.2298	2.03	623.2197 (12), 461.1676 (22), 161.0236 (100)	Echinacoside	X		
56	82.7		315.0506	C_16_H_11_O_7_	315.0510	1.26	300.0284 (100)	Quercetin methyl ether (Rhamnetin/isorhamnetin)		X	
57	82.9		345.0625	C_17_H_13_O_8_	345.0616	−2.80	330.0381 (100), 315.0162 (19)	Dimethylmyricetin		X	

* Compounds previously reported from the species investigated. X: compound present in the crude drug.

## Data Availability

All data generated or analyzed during this study are included in this article.

## Abbreviations

ABTS	2,2′-azino-bis(3-ethylbenzothiazoline-6-sulfonic acid)
CE	Catequin equivalent
DMAC	dimethylamino cinnamaldehyde
DPPH	2,2-diphenyl-1-picrylhydrazyl radical
FRAP	ferric reducing antioxidant power
GAE	Gallic acid equivalent
HPLC-DAD-MS/MS	high performance liquid chromatography coupled with diode-array detection and tandem mass spectrometry
ORAC	oxygen radical absorbance capacity
PAC	total proanthocyanidin
PEI	phenolic-enriched infusion
TE	Trolox equivalent
TEAC	Trolox equivalent antioxidant capacity
TF	total flavonoid
TP	total phenolic
